# Management of Mevalonate Kinase Deficiency: A Pediatric Perspective

**DOI:** 10.3389/fimmu.2020.01150

**Published:** 2020-06-05

**Authors:** Jerold Jeyaratnam, Joost Frenkel

**Affiliations:** ^1^Department of Obstetrics and Gynaecology, St. Antonius Hospital, Nieuwegein, Netherlands; ^2^Department of Pediatrics, Wilhelmina Children's Hospital, University Medical Center Utrecht, Utrecht, Netherlands

**Keywords:** mevalonate, autoinflammatory, hyperimmunoglobulinemia D syndrome, Interleukin-1, canakinumab, stem cell transplantation

## Abstract

**Background:** Mevalonate kinase deficiency (MKD) is an inborn error of metabolism leading to a syndrome characterized by recurrent inflammation. This clinically manifests itself as fever and can be accompanied by gastrointestinal symptoms, oral ulcers, cervical lymphadenopathy, and skin rash.

**Methods:** We searched Pubmed, Embase, Cochrane, and CINAHL for relevant articles. All articles were screened by both authors. Relevant articles were included in this review.

**Results:** The interleukin-1 antagonist canakinumab is the only well-studied and effective treatment for MKD patients with 35% of patients reaching complete remission in a large randomized controlled trial. Other therapeutic options include glucocorticoids and the IL-1 antagonist anakinra, although the level of evidence for these treatments is weaker. If patients fail to these treatments, the biologicals etanercept or tocilizumab can be used. Mildly affected patients might benefit from cheaper, less invasive treatments such as paracetamol and NSAIDs.

**Conclusion:** Canakinumab is the only evidence-based treatment for mevalonate kinase deficiency. However, the costs limit availability for many patients. Cheaper and more readily available options include glucocorticoids, anakinra, etanercept, and tocilizumab, although there is limited evidence supporting these treatments.

## Introduction

Mevalonate kinase deficiency is an autosomal recessive inborn error of metabolism. Bi-allelic mutations in the gene mevalonate kinase (*MVK)* lead to a reduced activity of the mevalonate kinase enzyme. This enzyme catalyzes the conversion of mevalonic acid to phosphomevalonic acid, an early step in isoprenoid biosynthesis. The defect leads to accumulation of its substrate, mevalonic acid, as well as to a shortage of isoprenoid end-products. Ultimately, these changes give rise to a syndrome, characterized by severe, more or less spontaneous recurrent inflammation. Patients suffer from episodic high-grade fever, that may be accompanied by oral ulcers, cervical lymphadenopathy, nausea, vomiting, diarrhea, skin rashes or arthritis. Complications include macrophage activation syndrome and ultimately AA-amyloidosis. Severely affected individuals may present with central nervous system damage or retinal degeneration, the pathogenesis of which is poorly understood. The spectrum of severity in mevalonate kinase deficiency is wide. The extremely rare, most severe phenotype is known as mevalonic aciduria (MA), whereas the more common, purely inflammatory phenotype had been known as the hyperimmunoglobulinemia-D periodic fever syndrome (HIDS) due to the elevated levels of serum immunoglobulin D in some of these patients (1). Understanding of the chain of events leading from the metabolic defect to the inflammatory phenotype is patchy. It is very likely that a shortage of certain isoprenoids, notably geranylgeranyl pyrophosphate leads to excessive release of interleukin-1β (IL-1β) (2). This appears to be mediated, at least in part, by inactivation of the small GTPase RhoA, and the subsequent activation of the Mediterranean fever (*MEFV*) gene product, pyrin (3). Pyrin, in turn, forms an inflammasome resulting in caspase-1 mediated IL-1β release. Since *MEFV* expression is limited to phagocytes, these cells are believed to be the source of inflammatory cytokines in mevalonate kinase deficiency. IL-1β exerts its pro-inflammatory effects through binding to the IL-1 receptor (2). All steps in this process are, at least in theory, amenable to therapeutic intervention. In addition to shortage of non-sterol isoprenoids, other potential mechanisms have been proposed, such as excess mevalonic acid, reduced 25-hydroxycholesterol and there are indications that cytokines other than IL-1β, such as gamma-interferon, TNF-α and IL-6 play an important role (4). In this paper we will address possible approaches and discuss the evidence for those that have been studied in clinical practice.

## Methods

We searched PubMed, EmBase, Cochrane and CINAHL for English language articles using the following search strategy: “mevalonate kinase deficiency” OR “hyperimmunoglobulinaemia d” OR “hyper igd” OR hids OR mvk OR “mevalonic aciduria”) AND (predniso^*^ OR canakinumab OR anakinra OR therapy OR treatment OR rilonacept OR glucocorticoid^*^ OR non-steroidal OR acetaminophen OR paracetamol OR colchicine OR simvastatin OR tocilizumab OR etanercept OR adalimumab OR infliximab. All articles were screened on title and abstract by both authors. After screening of title and abstract remaining relevant articles were screened on full-text. In addition, we reviewed the references of the retrieved papers for any missing sources. We did not exclude preclinical studies in order to address potential therapeutics avenues that have not (yet) reached clinical practice. In the analysis we rated studies according to the level of evidence provided (5).

## Results

**Figure 1:**
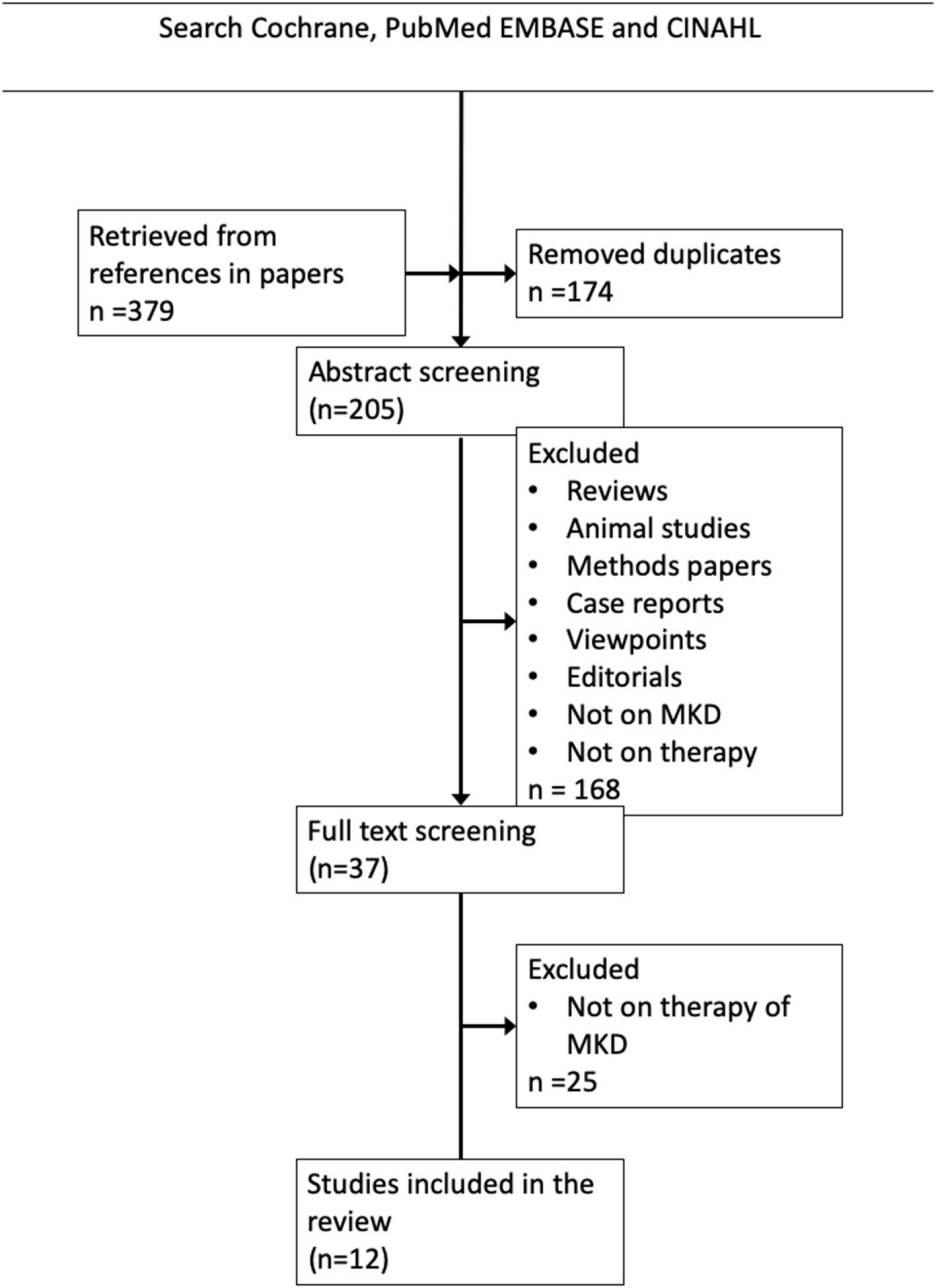
Literature search strategy and results. The Boolean search string in PubMed, Cochrane EMBASE and CINAHL was (((((((“mevalonic aciduria”) OR MVK) OR HIDS) OR “Hyperimmunoglobulinaemia D syndrome”) OR “Hyperimmunoglobulinemia D syndrome”) OR “mevalonate kinase deficiency”)) AND (((((((((((((acetaminophen) OR paracetamol) OR non-steroidal) OR simvastatin) OR glucocorticoid) OR predniso*) OR rilonacept) OR colchicine) OR canakinumab) OR anakinra) OR therapy)) OR treatment).

The search on Pubmed, Embase and Cochrane yielded 379 articles. After title and abstract screening 37 articles remained for full-text screening. Twelve articles were included for this review (Figure 1). We will discuss the findings along the pathophysiological chain from the genetic defect to the clinical symptoms.

### Gene Therapy

In theory, mevalonate kinase deficiency could be cured by repairing or replacing one of the mutant alleles in affected tissues. This would recapitulate the situation in heterozygous carriers, who are known to be clinically unaffected. However, to date, there have been no studies reporting such an approach, whether clinical or pre-clinical.

### Enzyme Replacement

Mevalonate kinase is a cytoplasmic enzyme. In contrast to lysosomal enzymes, exogenous replacement cannot restore the protein in its natural compartment. If excess mevalonate were responsible for the clinical features of the disorder, this would not matter very much. However, it is more likely that shortage of isoprenoid end products is to blame. No studies on enzyme replacement have been performed.

### Replacing the Affected Cells

Mevalonate kinase is a ubiquitous enzyme. However, the inflammatory phenotype most likely results from the expression of this defect in phagocytes. Therefore, replacing mutant myeloid cells by genetically normal ones by allogeneic stem cell transplantation, might be curative. No controlled studies have been published on this approach. Yet, several case reports have been published, some with encouraging results.

The first report described a 3-year old with mevalonic aciduria not responding to treatment with anakinra and etanercept. Allogeneic bone-marrow transplantation from an HLA-identical sister was performed. Follow-up after 15 months showed complete remission with normal inflammatory parameters, although the patients still had mild neurological manifestations (persistent atactic gait) (6). An report from the UK has described the same approach in an 8-year old boy with MA. This patient experienced severe graft-vs.-host disease after cessation of cyclosporine (due to inadequate chimerism) and suffered from several viral infections. All these problems resolved and at follow-up after 1 year the patient was in complete remission regarding febrile episodes (7). However, not all patients benefited from stem cell transplantation. A Turkish case-report described a boy with severe mevalonic aciduria leading to ascites and respiratory distress. After treatment with glucocorticoids and canakinumab had failed, an allogeneic bone marrow transplantation from an HLA-identical sister was performed at 138 days after birth. Although ascites regressed, the patient died due to septicemia 3.5 months after transplantation (8).

### Altering Isoprenoid Biochemistry

One small, randomized placebo controlled double blind cross-over trial with six patients studied reduction of the accumulating substrate mevalonate by means of oral statin treatment (9). There was a modest, but significant improvement in the treatment group with 5 of 6 patients reporting improvement while using simvastatin (level of evidence 1B). However, the largest retrospective series to date did not confirm these promising results, with statins failing in 11 of 15 patients. Three of these patients even mentioned worsening of their disease, while only four patients reported some benefit (level of evidence 3) (1).

Increasing geranyl-geranyl-pyrophosphate levels is a logical approach, which might be achieved by exogenous supplementation of geranyl-geranyl-pyrophosphate itself or its precursors or by skewing the metabolic flow in the mevalonate pathway toward geranyl-geranyl-pyrophosphate by blocking the enzyme squalene synthase. Both approaches have been investigated, but only in preclinical studies.

### Inhibiting the Pyrin Inflammasome

The pyrin inflammasome is central to the pathogenesis of another autoinflammatory disease, familial Mediterranean fever (FMF). Colchicine, which is an effective drug in FMF has not been formally studied in mevalonate kinase deficiency. Early reports noted its lack of efficacy (10). Retrospective data from the Eurofever registry indicate that colchicine may provide limited benefit in a minority of patients, but the evidence is weak (level of evidence 3) (1). Other inflammasomes, such as that containing NLRP3, might be involved in which case there would be a role for specific NLRP3-inhibtors. One pre-clinical study has investigated the link between the NLRP3 inflammasome and MKD in PBMC's from a mildly affected MKD patient. Stimulation of these cells with the NLRP3 specific stimulant nigericin led to much greater IL-1β production compared to PBMC's of either parent. When blocking the NLRP3 inflammasome by MCC950, this response was abolished. This suggests that blocking the NLRP3 inflammasome, might hold potential for the treatment of MKD (11). Four other NLRP3 inhibitors have been recently identified to be pharmacological inhibitors of the NLRP3 inflammasome. These inhibitors, CY-09, OLT1177, Tranilast, and Ordinonin have not been studied in a MKD or a model thereof. Two of these inhibitors (OLT1177 and Tranilast) appear to be relatively safe in humans who were treated for other conditions. However, further studies are needed to investigate the safety and therapeutic potential for MKD (12).

### Blocking Interleukin-1β

Since IL-1β plays an important role in the inflammatory phenotype of MKD, the IL-1β blocking agents anakinra and canakinumab have been employed in a substantial number of patients with success. The IL-1 antagonist rilonacept has not been described as a treatment for MKD.

A prospective, observational study including 11 MKD patients described the usage of anakinra (13). Patients with a more severe phenotype were assigned to continuous treatment, whereas patients with a milder phenotype were free to choose between continuous or on-demand treatment (children <16 years received 1.6 mg/kg/day, to be raised to a maximum of 2 mg/kg/day; adults received 100 mg/day, to be raised to a maximum of 200 mg/day in case of failure). This study showed that treatment was accompanied by shortening of fever attacks, lower CRP levels and reduction of symptoms, but did not lead to reduced frequency of fever episodes. This treatment appeared most effective when started within 24 h after the start of a fever episode. A disadvantage reported by patients using continuous treatment is the need of daily painful injections. Adverse events included local injection site reactions and upper respiratory tract infections (*n* = 2) (level of evidence 2B). Deshayes et al. described 10 adult patients receiving anakinra continuously (100 mg/day). Twenty percent failed to this treatment, while 50% responded partially. Another 30% had a complete response to anakinra. Though, two of the three patients with a complete response showed a loss of efficacy within 6 months after start of the treatment. Further, 60% suffered from injection site reactions, while 20% experienced respiratory infections (level of evidence 3) (14). The retrospective Eurofever registry reported continuous treatment with anakinra in 19 patients. Thirteen patients responded partially, while three patients had a complete response. Another three patients failed to this treatment. Eight patients used anakinra during attacks only, with three patients having a complete response and five a partial response (level of evidence 3) (1). Due to the retrospective design of this study, information about the dose was lacking. Failure to treatment might have been caused by inadequate dosing of anakinra.

Although anakinra has been beneficial for many patients, the long-term acting IL-1 antagonist canakinumab has been far better studied. In a large, randomized controlled trial (The CLUSTER trial) De Benedetti et al. investigated the efficacy of canakinumab in periodic fever patients, including 72 MKD patients (15). The included patients were ≥2 years old and had ≥6 attacks per year. This study showed that treatment with canakinumab, 150 mg (or 2 mg/kg in children <40 kg) every 4 weeks, leads to complete remission in 35% of patients after 16 weeks, compared to 6% in patients receiving placebo. Dose increase to 300 mg (or 4 mg/kg in children <40 kg) every 4 weeks further increased the effect to 57%. In a minority of patients, the dose could be subsequently reduced to 150 mg (or 2 mg/kg in children <40 kg) every 8 weeks. Patients who did not achieve complete remission still had a significant reduction in attack frequency. Patients receiving canakinumab also scored better on a physician's global assessment score compared to placebo and had a significant reduction of C-reactive protein levels. During this trial, there were no reported deaths or opportunistic infections such as tuberculosis. Seven serious adverse events were reported while using canakinumab (three cases of pneumonia and one case of pharyngitis, laryngitis, gastroenteritis and conjunctivitis) (level of evidence 1B). Another study described seven patients being treated with canakinumab. Four of them had a partial response, while three had a complete response. Two patients who responded partially had failed to respond to anakinra earlier (level of evidence 3). This long acting treatment only requires monthly injections, which is a major advantage for patients compared to daily injections when using anakinra. However, the cost of this drug (≥$70,000/year) preclude its use by many patients. In comparison, anakinra costs ~$35–40 for a single dose of 150 mg (annual costs depend on continuous or on-demand use, but in Western countries canakinumab is ~7–10 times more expensive).

### Blocking Other Cytokines

Since TNF-α and IL-6 are thought to contribute to the pathophysiology of MKD, antagonists of these cytokines have been used to treat patients.

TNF blockade with etanercept has been beneficial in a number of patients. Etanercept has been described in a retrospective case series in 27 patients, with 16 patients reporting some benefit and 11 patients not responding (16). Two of these 16 patients reached complete remission (level of evidence 3). Another retrospective study described 8 patients assigned to etanercept, with 7 of them achieving complete remission on this treatment (level of evidence 3) (1). However, another retrospective study reported failure of etanercept in 5 out of 9 patients (level of evidence 3). Given the superior evidence for IL-1 blockade, etanercept is nowadays not a first-choice option, but mostly used to treat patients who have no access to or no response on IL-1 blockade (level of evidence 3).

Tocilizumab is not widely used to treat patients with MKD. However, it has been used successfully in several patients who did not respond to treatment with other biologicals or glucocorticoids. Treatment with tocilizumab has been described in a case of a 13-year old girl after failing to anakinra and etanercept. On this treatment her disease went in remission, although infections had been an initial concern (level of evidence 4) (17). Another case report has mentioned a 32-year old woman failing treatment with simvastatin, NSAIDs and anakinra leading to hospitalization 11 times in 1 year (18). Treatment with tocilizumab was started along with methylprednisolone, since treatment with tocilizumab was experimental. This treatment was followed by remission, which enabled tapering of methylprednisolone. Further, a retrospective study described two MKD patients with a complete response to tocilizumab. Prior to this treatment they failed to anakinra and/or etanercept. One of this patients underwent kidney transplantation due to AA-amyloidosis. After this transplantation he developed EBS septicemia, which led to halving of the tocilizumab dose until the septicemia resolved (19).

### Non-specific Inhibition of Inflammation

Treatment with paracetamol and non-steroidal anti-inflammatory drugs (NSAIDs) can relieve symptoms during inflammatory attacks, but does not lead to remission. Although evidence for this approach is lacking, symptomatic treatment of disease flares this way is standard practice and may be sufficient to control mild infrequently relapsing disease. Glucocorticoids also reduce symptoms in MKD patients, especially when given in high dose at the start of an attack (20).

#### Other Therapies

In severely affected patients with ongoing inflammation, the disease can lead to AA-amyloidosis and ultimately kidney failure. The Eurofever cohort reported five patients with AA-amyloidosis (in a cohort of 114 patients). Four of these patients underwent kidney transplantation due to end-stage kidney failure, while one patient died due to the complications of dialysis (1). Therapy with biologicals can be continued in the work-up period toward kidney transplantation when patients are undergoing hemodialysis.

## Recommendations

Given the dearth of evidence, any recommendation will ultimately be based on expert opinion and can be of limited strength only. The recommendations below are given by pediatricians.

Symptomatic relief during attacks, though never formally studied, is provided by non-steroidal anti-inflammatory agents and paracetamol (evidence level 5, strength of recommendation D). The low costs of these drugs enable treatment for a large group of mildly affected patients with a limited number of fever attacks.

The only truly evidence-based therapy in mevalonate kinase deficiency is IL-1 blockade with canakinumab (evidence level 1b). It is the treatment of choice in patients with frequent disease flares (strength of recommendation A). However, canakinumab is very costly and hence not available to many patients. Moreover, the study design had excluded young (<2 year old) patients and those with infrequent disease flares limiting the evidence to older and more severely affected individuals. Anakinra (evidence level 3) is a rational alternative as it is less expensive, but many patients don't have access to it either or do not tolerate the daily painful injections (strength of recommendation C). However, pain at the injection site can be reduced by local application of ice packs before or by hydrocortisone cream after injection. In patients in whom IL1 antagonists are not tolerated or ineffective, maintenance therapy with tocilizumab or etanercept may be attempted (evidence level 4, strength of recommendation D). In patients with uncontrollable diseases who have no access to or do not respond to biologicals, empirical treatment with high dose glucocorticoids may be attempted.

Continuous cytokine blockade is not warranted in patients with infrequent attacks who recover fully in-between. In such patients we advise treatment on demand to abort attacks. The level of evidence supporting any of these strategies is low (strength of recommendation D).

**Figure 2:**
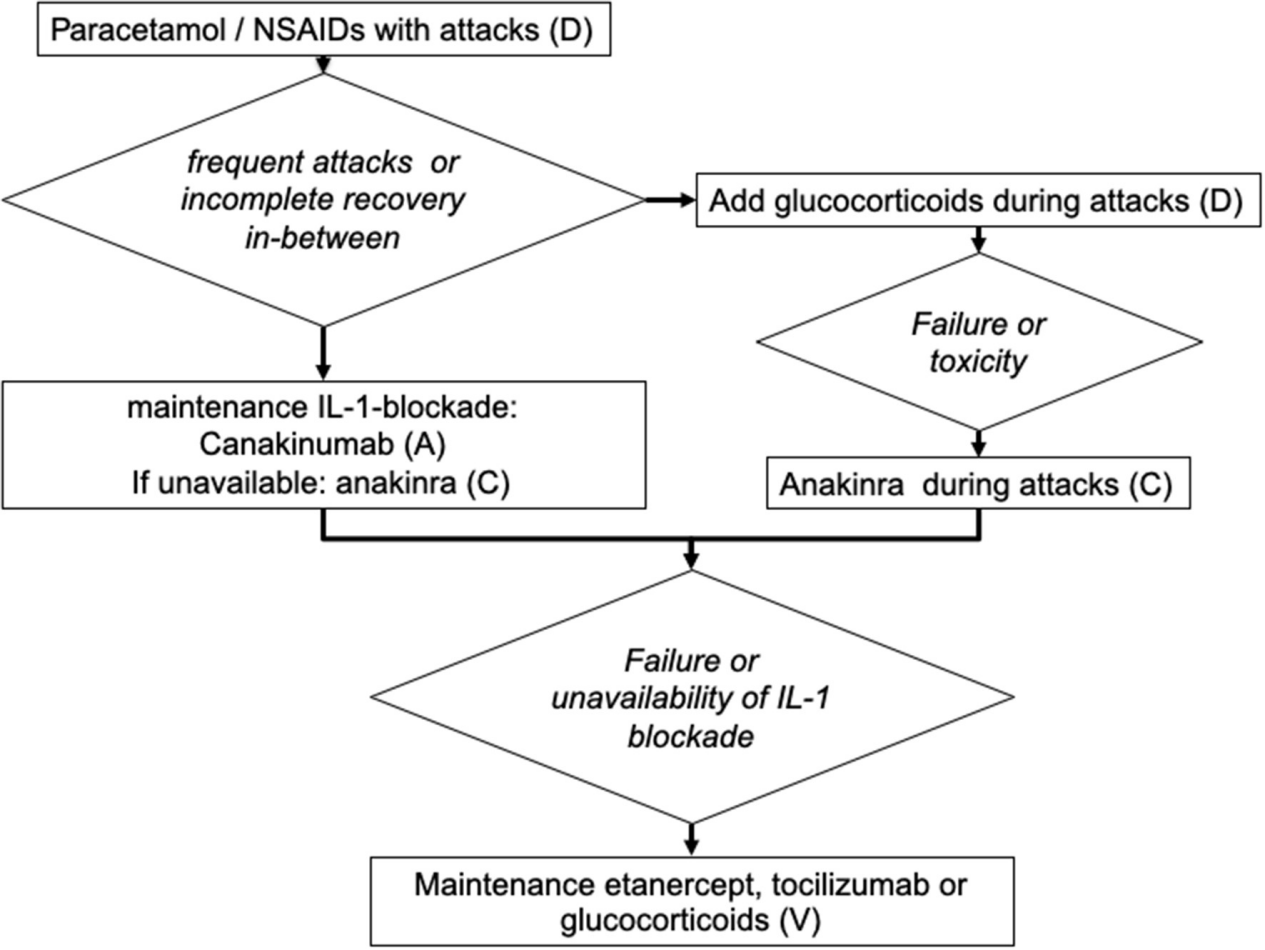
Suggested algorithm for the treatment of patients with mevalonate kinase deficiency (adapted for the treatment of children). The capital letters (A–D) indicate the strength of recommendation (5).

We have summarized these recommendations in a tentative flow diagram (Figure 2).

None of these approaches addresses the basic molecular defect. Future avenues might do so. These include gene therapy and supplementing of deficient isoprenoid metabolites. They hold promise, however uncertain, of respectively, cure or disease control by oral medication.

## Conclusions

There is strong evidence for the effectiveness of canakinumab in mevalonate kinase deficiency. The design of the CLUSTER study excluded the most severely affected patients (those with intractable disease before the age of 2 years) and mildly affected patients. The latter may benefit from cheaper, less invasive measures, such as NSAIDS or glucocorticoids. When treatment with canakinumab is unavailable, anakinra is a rational alternative. When IL-1 blockade fails or is unavailable, treatment with other biologicals, such as etanercept or tocilizumab, can prove to be successful. Only in the severest of cases should allogeneic stem cell transplantation be considered.

## Author Contributions

The search strategy and draft of the article were done by JJ and JF.

## Conflict of Interest

JF received consultancy fees from Novartis. The remaining author declares that the research was conducted in the absence of any commercial or financial relationships that could be construed as a potential conflict of interest.
